# Using Genomics to Design a Pathovar-Specific Loop-Mediated Isothermal Amplification (LAMP) Assay, for the Improved Detection of *Xanthomonas citri* pv. *citri*

**DOI:** 10.3390/microorganisms10061153

**Published:** 2022-06-02

**Authors:** John Webster, Monica A. Kehoe, Elisse Nogarotto, Linda Falconer, Nerida Jane Donovan, Toni A. Chapman

**Affiliations:** 1 NSW Department of Primary Industries, Elizabeth Macarthur Agricultural Institute, Menangle, NSW 2568, Australia; linda.falconer80@gmail.com (L.F.); nerida.donovan@dpi.nsw.gov.au (N.J.D.); toni.chapman@dpi.nsw.gov.au (T.A.C.); 2 DPIRD Diagnostics and Laboratory Service, Department of Primary Industries and Regional Development, South Perth, WA 6151, Australia; monica.kehoe@dpird.wa.gov.au; 3 AgriBio, Centre for AgriBioscience, Agriculture Victoria Research, Melbourne, VIC 3083, Australia; elisse.nogarotto@agriculture.vic.gov.au

**Keywords:** loop mediated isothermal amplification (LAMP), *Xanthomonas*, Citrus Canker, rapid diagnostic

## Abstract

The ability to swiftly respond to pathogen incursions relies heavily on fast and accurate diagnostics. Current published assays for citrus bacterial canker do not target *Xanthomonas citri* pv. *citri*, the causative agent, with high specificity when testing Australian samples. While the current diagnostics are useful in countries where canker is endemic, the detection of canker in Australia requires an emergency response. Close relatives to *X. citri* pv. *citri* found in Australia may generate false positives with the current recommended diagnostic assays. Therefore, we developed a more specific detection tool for citrus bacterial canker to provide greater diagnostic confidence for surveillance and eradication efforts. We used genomic comparisons of 161 Xanthomonad genomes and identified and confirmed genomic regions specific for *X. citri* pv. *citri* by performing local alignments of unique regions to reference genomes. We then developed loop-mediated isothermal amplification primers and validated them against a panel of 190 isolates to confirm specificity. Our diagnostic assay showed 100% corroboration with the concurrently developed multiplex primers and represents an improved diagnostic method capable of effective citrus bacterial canker identification.

## 1. Introduction

Citrus bacterial canker (CBC) is a serious disease, causing unsightly lesions on fruit, leaves and stems, and reduced yield through premature fruit drop and the downgrading of harvested fruit [1]. Originating in Southeast Asia, the disease has spread to many tropical and sub-tropical regions, affecting many commercial varieties. There are proven management options by which to reduce the impact of CBC including windbreaks, copper-based bactericides, decontamination of equipment, control of leaf miner, and use of tolerant or resistant varieties [2,3,4]. However, the Australian citrus industry considers CBC to be a major emergency plant pathogen due to the potential crop loss and increased cost of production associated with managing this highly contagious disease.

There have been several recorded incursions of CBC in Australia with the most recent detections in the Northern Territory (NT) in April 2018 [5], North-west Western Australia (WA) in 2018 [6] and in Emerald, Queensland in July 2004 [7]. Australia’s biosecurity preparedness for these types of disasters led to the eradication of CBC from Emerald before it could become widespread, thus saving the industry an estimated $70 Million [7]. It was also declared eradicated from WA in November 2019 [6], and from the NT in April 2021 [8]. Effective detection tools are essential to aid surveillance and eradication responses, prevent wide scale infection, and avoid subsequent economic losses in the event that CBC becomes established in Australia.

Detection tools for CBC target the bacterium *Xanthomonas citri* pv. *citri* (Xcc), the causative agent of CBC and include methods such as culturing bacteria from suspect cankers and re-inoculating a citrus host, conventional polymerase chain reaction (PCR), Sanger sequencing, and more recently, loop-mediated isothermal amplification (LAMP) [9,10,11]. Of these methods, LAMP does not require lengthy culture processes and is more efficient than conventional PCR, providing an obvious advantage when dealing with incursions in new areas. Additionally, LAMP assays may be performed at the site of suspected CBC finds with minimal effort. This can be achieved using lateral flow dipstick designs, such as those used for other phytopathogens [12], as well as with field deployable LAMP machines, such as the Genie^®^ (Optigene, Horsham, UK).

Diagnostic methods need to be fast and reliable to maintain this level of success seen in Australia. Currently, the Australian National Diagnostic Protocol (NDP) recommends diagnostic assays targeting the pth-A gene by Hartung et al. (1993) [13], Cubero and Graham (2002) [14] and Mavrodieva et al. (2003) [11]. Published molecular diagnostics have resulted in false positives with endemic Xanthomonads in Australia. This is also true for the LAMP assay designed by Rigano et al. [9] which cross reacts with *X. citri* pv. *malvacearum*, the causative agent of bacterial blight of cotton. This is an issue in some regions in Australia where cotton and citrus are grown in adjacent fields and *Xanthomonas citri* pv. *malvacearum* is ubiquitous in the cotton growing regions of Australia. False positive results potentially confound eradication efforts. Therefore, we designed a LAMP assay using genome sequencing data to target *X*. *citri* pv. *citri* with high exclusivity of non- *X*. *citri* pv. *citri* isolates and improve Australian biosecurity preparedness for CBC.

## 2. Materials and Methods

### 2.1. Identifying Pathovar Specific Regions for X. citri pv. citri

We used *X. citri* pv. *citri* UI6 as a *X. citri* pv. *citri* representative, and we made genomic comparisons against a large concatenation (157 genomes, Supplementary Table S1) of non *X. citri* pv. *citri* genomes using the ‘Uniqprimer’ function in the South Green Bioinformatics platform (http://galaxy.southgreen.fr/galaxy/, accessed on 8 March 2018).

The unique regions produced by ‘Uniqprimer’ were compared against a concatenated dataset of genomes that included *X. citri* pv. *mangiferaeindicae* (DAR82810), *X. fuscans* pv. *fuscans* strain 4834R and *X. citri* pv. *glycines* (DAR82581), using the same ‘Uniqprimer’ function. The remaining unique regions were filtered for hits that matched *X. citri* pv. *citri* genomic regions by using BLASTn against the NCBI database. Sequences with only hits to *X. citri* pv. *citri* were locally aligned in Geneious to the set of negative genomes above, and additionally *X. fuscans* pv. *aurantifolii* 1622, using the Geneious read mapping algorithm, for *X*. *citri* pv. *citri* specificity. We assumed that sequences that failed to map to negative genomes were unique to *X. citri* pv. *citri*, and LAMP primers were then designed using Primer Explorer V5 (Table 1). As a final check, BLASTn was used for LAMP primers against the NCBI database and locally aligned in Geneious to the excluded genomes to assess suitability. Additionally, unique regions were examined in IMG/ER (https://img.jgi.doe.gov/cgi-bin/mer/main.cgi, accessed on 25 March 2018) to determine the gene regions of the *X. citri* pv. *citri* genome for which primers were designed.

### 2.2. LAMP Reaction

The LAMP assay was performed in 25 μL reaction volumes with a Rotor-gene Q Real Time PCR machine (Qiagen, Hilden, Germany) using Tin(exo-) Isothermal Mastermix (Optigene, Horsham, UK) at a 1× concentration and 1.6 μM of both FIP and BIP primers, 0.2 μM of outer primers F3 and B3, and 0.4 μM of loop primers LF and LB. The reaction profile consisted of an initial 95 °C denaturation for 5 min, followed by isothermal amplification at 65 °C for 30 min. Primers were sent to two alternate laboratories for testing on a Genie^®^ III (Optigene, Horsham, UK) with GspSSD2.0 Isothermal Mastermix (Optigene, Horsham, UK) at 1× concentration and the primer concentrations mentioned above. The limit of detection was determined by a 10-fold dilution series between 1 ng/μL and 10 fg/μL, quantified with a Qubit High Specificity kit (Thermofisher Scientific, Waltham, MA, USA). Primers were validated against a panel of 190 DNA samples of 21 *X. citri* pv. *citri*, 20 *X. citri* pv. *malvacearum*, 5 *X. citri* pv. *mangiferaeindicae*, 88 endemic Xanthomonads (including *X. arboricola*, *X. axonopodis* pv. *phaseoli*, *X. campestris*, *X. campestris* pv. *pruni*, *X. campestris* pv. *sesame*, *X. campestris* pv. *vesicatoria*, *X. citri* pv. *phaseoli* var. *fuscans*, *X. gardneri*, *X. hortorum*, *X. oryzae*, *X. perforans*, *X. translucens*, *X. vasicola* and *X. vesicatoria*), 36 citrus leaf samples (non-CBC infected) from citrus-producing regions across Australia encompassing different climatic zones, 10 healthy cotton samples (leaves) from different farms in Northern NSW and 10 healthy mango samples (leaves) from Queensland. 

### 2.3. DNA Extraction

Extractions of DNA were performed directly from colonies of known *Xanthomonas* spp. Using a Qiagen Dneasy Blood and Tissue kit (Qiagen). Lesions from both healthy leaf material and CBC diagnostic leaf samples collected during the Northern Territory biosecurity response were macerated in 400 µL of sterile water using sterilised scissors and left for 30 min to allow bacterial streaming from the lesions to occur. This extract was then processed with the Dneasy Plant Mini kit (Qiagen) according to the manufacturer’s protocols. 

### 2.4. PCR Detection Methods

Samples were additionally tested using two alternative PCR methods, a concurrently designed multiplex assay (Toni Chapman pers. Comm.) and the Jpth assay by Cubero and Graham (2002), targeting the pthA gene. The Jpth assay was performed as per the specifications as outlined by the Australian National Diagnostic Protocol for the detection of *X. citri* pv. *citri* (https://www.plantbiosecuritydiagnostics.net.au/app/uploads/2018/11/NDP-9-Asiatic-citrus-canker-Xanthomonas-V1.2.pdf, last accessed on 25 May 2022). 

## 3. Results

LAMP primers were validated against a panel of 190 samples of known isolates, diagnostic samples and leaves from citrus, mango, and cotton. Multiple sets of primers passing all filtering criteria were tested and XccLAMP219 primers were the only set that showed 100% inclusivity LAMP results for *X. citri* pv. *citri* isolates and exclusivity for closely related organisms (Table 2).

### 3.1. Limit of Detection

Serially diluted *X. citri* pv. *citri* DNA was reliably identified at the lowest concentration of 80 fg/uL for the final reaction, equating to a theoretical number of cells of ~14 per μL.

### 3.2. Genomic Region

Gene neighbourhoods identified in IMG/MER show that the *X. citri* pv. *citri* genome region for which the LAMP assay was designed was within a region coding for a type IV secretion system (T4SS) element. The alignment of the LAMP region to *X. citri* pv. *citri* 306 further identified the LAMP assay region belonging to a short fragment (180 bp) of the DotA/TraY protein family (Supplementary Figure S1). This coding region was only present in *X. citri* pv. *citri*, *X. citri* pv. *malvacearum*, *X. euvesicatoria* and *X. oryzae* pv. *oryzae*, the complete level genomes in IMG/MER. However, the region in which the LAMP was designed was not present in any other isolates apart from *X. citri* pv. *Citri* (Supplementary Figure S2). 

### 3.3. Incursion Response

During design of this diagnostic, a new incursion of CBC in Australia provided an opportunity to validate our LAMP assay in parallel with the Jpth assay [13], as specified in the approved National Diagnostic Protocol. The LAMP assay designed here confirmed all positively identified isolates strengthening diagnostic confidence during a critical phase of the incursion. Further optimisation revealed the capability of the test to be performed on crude maceration extracts from suspect canker lesions and from bacterial isolates without the need for DNA extractions. This allowed for testing to be completed within an hour of sample receipt and proved to be useful for providing provisional results for important biosecurity samples before culturing, isolation and DNA extractions were complete. Due to the high specificity of this assay, a sample that had produced a false positive using the National Diagnostic Protocol was identified. The false positive sample was identified by a diagnostic lab during the incursion response and produced an amplified product using the Hartung et al. 1993 assay. Sequencing of the PCR amplicon returned a result of *X. citri* pv. *citri*. However, the Jpth assay also produced no product and 16S identification was required to confirm that the isolate was a Hartung false positive, *Pantoea dispersa*.

## 4. Discussion

Published LAMP identification methods are unable to distinguish between *X. citri* pv. *citri* and *X. citri* pv. *malvacearum*. Diagnostic tests that are not specific for *X. citri* pv. *citri* may be confounded by *X. citri* pv. *malvacearum* or other Xanthomonads. The likelihood of cross-reacting Xanthomonads existing on citrus canker lesions may be low, and a thorough examination of suspect lesions for consistency with canker and bacterial ooze further reduces the risk of false positives. However, false positive detections have occurred in Australia, therefore generating the need for an improved method. Other previously published diagnostic methods for CBC have also been observed to produce positive results for non-target strains [15]. In the study by Delcourt et al. 2013, Jpth primers, among others, were observed to produce positive assay results when tested against *X*. *citri* pv. *aurantifolii*, *X. citri* pv. *bilvae* and ‘other’ pathogenic *Xanthomonas* species. The European and Mediterranean Plant Protection Organization (EPPO) recommend the use of several sets of primers, including J-pth1/2, J-Rxg/c2, 2/3 and 47; all of which were also identified by Delcourt et al. to show less than 100% inclusivity of *X. citri* pv. *citri* or exclusivity of other Xanthomonads when tested against various isolates. We designed a highly specific diagnostic assay tailored for the identification of *X. citri* pv. *citri*, producing no false positives with other closely related *Xanthomonas* spp. 

This was achieved using sequencing techniques such as whole genome Illumina sequencing and analyses to determine specific genomic regions of *X. citri* pv. *citri*. The target gene region where LAMP primers were designed revealed the presence of a Type 4 Secretion system (T4SS) element, DotA/TraY family protein (locus tag XAC_RS12305, *X. citri* pv. *citri* UI6). Type IV secretion systems can be classified into the following three types: IVA, IVB and ‘Other’ [16]. These systems are complexes of proteins that span the cell envelope and allow bacteria to translocate proteins and DNA–protein complexes into other cells, such as in the case of T4ASSs, or can facilitate conjugation, such as T4BSSs [16]. Other Gram-negative bacteria are known to use T4SS as a key virulence factor in pathogenicity [17,18], and the involvement of T4SS in *X. citri* pv. *citri* has been linked with biofilm development. In addition, a mutant of XAC3266, which previous studies suggest interacts with VirD4 of the T4SS [19], was observed by Malamud et al. to show a significant decrease in symptoms of *X*. *citri* pv. *citri* in infected leaves [20], implicating the T4SS in *X*. *citri* pv. *citri* infection. The coding region identified in this study, DotA/TraY, belongs to T4BSSs and was associated with the conjugal transfer of plasmids as well as survival in its host for *Legionella pneumophila* [21] and was also previously observed in plasmid pBX01-1 of *X. oryzae* pv. *oryzae* [22].

Sequencing technologies can be used to identify genomic regions that may play pivotal roles in virulence. The identification of these regions in silico opens up the possibility for targeted diagnostic assays, as seen here. Robène et al. (2020) also performed similar genomic searches whereby they compared 30 X. *citri* pv. *citri* genomes against 30 non-target *Xanthomonas* to identify coding regions that were present in only *X. citri* pv. *citri* and showed no or low identity to non-target genomes [23]. They identified a CDS, XAC1051, and were able to generate a conventional PCR (XAC1051-F/R) that targeted *X. citri* pv. *citri* exclusively, and a real time assay (XAC1051-2qPCR) that showed limited cross reactivity. 

Published methods are available for CBC but the results observed in our study were achieved quickly using LAMP technology, producing an accurate result within 30 min from DNA extraction. The LAMP primers used in a real-time PCR assay provide a means for the high-throughput laboratory testing of surveillance samples, either pre- or post-incursion. There is potential for this LAMP assay to be incorporated into a lateral flow device providing a portable diagnostic solution. Field-deployable technologies allow for a fast turn-around time from initial observation to the identification of suspect CBC. The ability to test suspect lesions on plants in the field, and the testing of potentially contaminated farm equipment, allows for decisions to be made earlier during CBC outbreaks, increasing the likelihood of successful containment and eradication. Where CBC is endemic, a portable diagnostic would allow for the testing of nursery trees prior to orchard establishment as part of an integrated disease management program. Regardless of whether methods are used in the field or the laboratory, LAMP has a faster turn-around time and the specificity delivered in this new assay will be beneficial for the detection of new incursions and management of CBC.

## Figures and Tables

**Table 1:** LAMP primers designed on *X. citri* pv. *citri* unique regions.

Primer Name	Type	Sequence (5′–3′)	Length
XccLAMP219-F3	F3	CCCACGGCTACATCTTCCT	19 mer
XccLAMP219-B3	B3	TGCACAAGGTTGAGACACAT	20 mer
XccLAMP219-FIP	FIP (F1c + F2)	GTTCCGCCTGCGATGACTCC-CTTGGAGATGATGGTGCGT	39 mer
XccLAMP219-BIP	BIP (B1c + B2)	GTTGCTGAACGAGGGGTTCGA-AGGCCAGAATCGAACCGAT	40 mer
XccLAMP219-LF	LF	CGAGCACCATGAGCACAGG	19 mer
XccLAMP219-LB	LB	CATTGCCCTTGCAAACGCT	19 mer

**Table 2:** Results of LAMP primers designed for *X. citri* pv. *citri* specificity compared to the *X. citri* pv. *citri* multiplex and Jpth assay.

Sample Type	Number of Jpth Positives ^	Number of Multiplex Positives	Number of LAMP Positives (This Study)
*X. citri* pv. *citri*	19/19	21/21	21/21
*X. citri* pv. *malvacearum*	20/20	0/20	0/20
*X. citri* pv. *mangiferaeindicae*	5/5	0/5	0/5
Other Endemic Xanthomonads	18/21	0/88	0/88
Citrus leaves (non-CBC)	0/20	0/36	0/10
Cotton Leaves (uninfected)	10/10	0/10	0/10
Mango Leaves (uninfected)	ND	0/10	0/10

ND = Not Determined; ^ Jpth tested against a smaller sample subset.

## Data Availability

Not applicable.

## Supplementary Materials

The following supporting information can be downloaded at: https://www.mdpi.com/article/10.3390/microorganisms10061153/s1, Table S1: List of isolates used for genome comparisons, Figure S1: Gene target region for XccLAMP219 against reference genome *Xanthomonas citri* pv. *citri* str. 306 (NC_003919.1), Figure S2: Gene alignment of all Xanthomonas ‘Finished’ level genomes on IMG/MER that contained a copy of the DotA/TraY coding region. XccLAMP219 region is annotated on *X*. *citri* pv. *citri* 306.
